# Increase in tropical cyclone rain rate with translation speed

**DOI:** 10.1038/s41467-022-35113-8

**Published:** 2022-11-28

**Authors:** Shifei Tu, Johnny C. L. Chan, Jianjun Xu, Quanjia Zhong, Wen Zhou, Yu Zhang

**Affiliations:** 1 China Meteorological Administration-Guangdong Ocean University (CMA-GDOU) Joint Laboratory for Marine Meteorology & South China Sea Institute of Marine Meteorology, Guangdong Ocean University, Zhanjiang, China; 2 College of Ocean and Meteorology, Guangdong Ocean University, Zhanjiang, China; 3 Shenzhen Institute of Guangdong Ocean University, Shenzhen, China; 4 Low-Carbon and Climate Impact Research Centre, School of Energy and Environment, City University of Hong Kong, Hong Kong, China; 5 Shanghai Typhoon Institute, China Meteorological Administration, Shanghai, China; 6 Asia-Pacific Typhoon Collaborative Research Center, Shanghai, China; 7 State Key Laboratory of Numerical Modeling for Atmospheric Sciences and Geophysical Fluid Dynamics (LASG), Institute of Atmospheric Physics, Chinese Academy of Sciences, Beijing, China; 8 Department of Atmospheric and Oceanic Sciences, Institute of Atmospheric Sciences, Fudan University, Shanghai, China

**Keywords:** Atmospheric science, Natural hazards

## Abstract

In general, tropical cyclone (TC) rainfall accumulation usually decreases with faster TC translation speed but increases with heavier rain rate. However, how the TC rain rate changes with translation speed is unclear. Here we show that, in all TC basins, the average TC rain rate significantly increases with translation speed. On average, the rain rate in a fast-moving TC is 24% higher than in a slow one. This difference increases with TC intensity, with category 3–5 TCs having a 42% increase while tropical depressions exhibit only a 9% increase. The increase in the average TC rain rate with translation speed is mainly caused by the TC net inflow in the lower troposphere, as well as vertical wind shear. These findings have important implications not only for a deeper understanding of rain rate changes in a translating TC but also for short-term forecasts of TC rainfall and disaster preparedness.

## Abstract (web summary)

Using satellite observations, the authors show that the average tropical cyclone (TC) rain rate increases significantly with translation speed. On average, the rain rate of a fast-moving TC is about 24% higher than that of a slow one.

## Introduction

Tropical cyclones (TCs) usually generate over the tropical and subtropical oceanic regions, and subsequently migrate westward and poleward under the combined effects of the atmospheric steering flow and the β effect^1,2^, quite often threatening coastal areas. In recent decades, the locations of TC maximum intensity (mostly in the western North Pacific, eastern North Pacific, and South Indian Ocean) appear to be migrating towards the coasts^3^ and the polar regions^4^, which means that TC-related threats over the coastal areas and higher latitudes may increase. This fact can be partially supported by statistics from the World Meteorological Organization, which shows that TC-related disasters and economic losses have increased in recent decades^5^.

In general, the potential threat posed by a TC is related to its intensity^6,7^, which is affected by the TC translation speed^8^. Slower-moving TCs tend to generate stronger sea surface cooling and have longer exposure to the cooling, both of which tend to strengthen the negative SST feedback, then decrease the TC intensity. Therefore, TC intensity increases with translation speed, but it is worth noting that the very fast-moving TCs are not conducive to the maintenance of TC symmetrical structure, thereby inhibiting TC intensification^8^. An ideal TC numerical simulation shows a translation speed of 3 m s^–1^ to be the optimal speed for TC development when the mixed-layer depth is thick enough^9^.

Heavy rainfall induced by TCs is one of the important factors causing disasters, such as floods, urban waterlogging, landslides, and mudslides^5,10,11^. Generally, TC rainfall-related disasters are primarily caused by high TC rainfall accumulation, which is mainly driven by the average TC rain rate and translation speed^12–14^. Specifically, a slow-moving TC usually has a larger TC rainfall accumulation because of the longer TC influence time, and an increase in TC rain rate can also lead to an increase in TC rainfall accumulation. However, it is still unclear whether a significant relationship exists between TC rain rate and translation speed because most previous studies explored the characteristics of TC rain rate and translation speed separately. The existence of such a relationship has important implications not only for a deeper understanding of rain rate changes in a translating TC but also for short-term forecasts of TC rainfall and disaster preparedness.

Here we find a significant linear relationship between the average TC rain rate and the translation speed, where the faster-moving TCs usually be accompanied by the larger TC rain rates. This phenomenon is synergistically affected by the TC net inflow and vertical wind shear.

## Results

### Tropical cyclone rain rate changes with translation speed

**Fig. 1:**
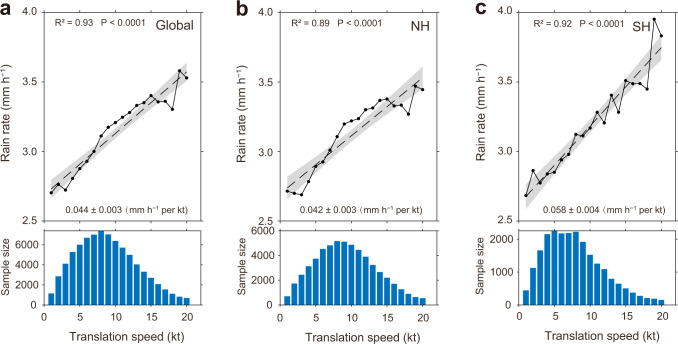
Changes in average tropical cyclone (TC) rain rate with translation speed. **a** Global, **b** Northern Hemisphere (NH), **c** Southern Hemisphere (SH). Only the rainy-pixels average (see Methods) is shown here. The results of the all-pixels average are shown in Supplementary Fig. 2a–c. The shaded area in all panels indicates the two-sided 95% confidence levels of the slopes, and the dashed lines represent the linear regression of the rain rate with TC translation speed. All linear trends are significant at the 99% confidence level. The bar chart in each sub-figure represents the distribution of TC samples.

Here we show that, globally, the average TC rain rate increases significantly with translation speed (Fig. 1a), with a rate (hereafter the “growth rate”) of 0.044 ± 0.003 mm h^–1^ per kt (Supplementary Table 1, note: 1 kt ≈ 0.514 m s^–1^ ≈ 1.852 km h^–1^). This result is based on a systematic study on the relationship between TC rain rate and translation speed using high-resolution and real-time global rainfall observations from the Integrated Multi-satellite Retrievals from the Global Precipitation Measurement (IMERG) final precipitation dataset^15^ for the period 2001 to 2020 (see Methods, Supplementary Fig. 1). The TC best-track dataset is from the International Best Track Archive for Climate Stewardship (IBTrACS)^16^. Similar results are obtained for TCs in the northern and southern hemispheres (Fig. 1b, c).

To validate these results further, the TC rain rates from the Tropical Rainfall Measuring Mission (TRMM) Multi-satellite Precipitation Analysis (TMPA) dataset^17^ as well as the fifth generation ECMWF (European Centre for Medium-Range Weather Forecasts) atmospheric reanalysis (ERA5) dataset^18^ are also examined (Supplementary Fig. 2d, e). This increase in rain rate with translation speed is also apparent although growth rates are slightly different because of the uncertainties in different datasets^19,20^. The phenomenon that the average TC rain rate increases with translation speed are therefore robust. Further, this phenomenon exists in all six TC basins, TC categories, latitudinal belts, and over the ocean and land (Supplementary Table 1, Supplementary Fig. 2f–u). In addition, the rain rate in fast-moving (16–20 kt) TCs has a 24% increase from the slow-moving (1–5 kt) TCs (Supplementary Table 1). This difference also increases with TC intensity, with a 9% increase for tropical depressions and a 42% increase for category 3–5 TCs.

Because rain rates span a very large range within TCs (usually including weak, moderate, and heavy rain), how do these different types of rain intensities change with translation speed? On a global scale, the probability of weaker rain rates (<1 mm h^–1^, mostly the weak rain) decreases with TC translation speed, but that of the higher rain rate (3~40 mm h^–1^, mostly the moderate and heavy rain) increases (Supplementary Fig. 3a). This phenomenon also exists for both hemispheres, different TC intensities, and in different TC basins (Supplementary Fig. 3b–m). In other words, the significant increase in the average TC rain rate with translation speed is driven by the increased probability of moderate and heavy rate rates.

### Inflow and tropical cyclone rain rate

**Fig. 2:**
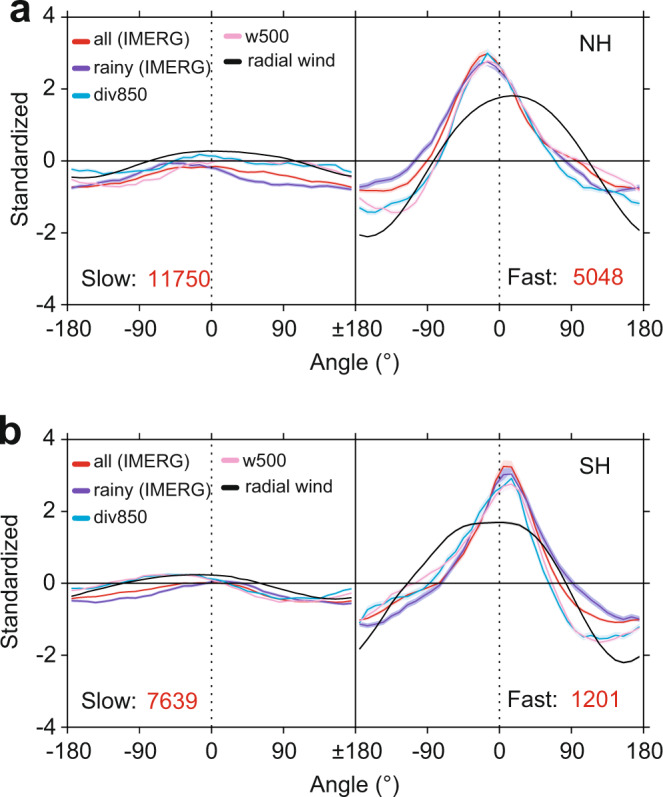
Azimuthal distribution of different standardized variables in the two hemispheres. Left and right panels in each sub-figure represent these variables in slow- and fast-moving tropical cyclones (TCs), respectively. **a** Northern Hemisphere (NH), and **b** Southern Hemisphere (SH). The *x* axis represents different angles, as in the schematic diagram shown in Supplementary Fig. 1c; “0” represents the direction of TC motion, “±180” represents the reverse of TC motion, and clockwise is positive. Colors are for different standardized variables: all-pixels average (red) and rainy-pixels average (purple) in TC rain rate, vertical velocity at 500 hPa (pink, upward is positive), divergence at 850 hPa (blue, convergence is positive), and radial wind at 10 m (black, inflow is positive). Red numbers indicate the sample sizes used in each plot.

To identify the possible mechanism for the increase in average rain rate in fast-moving TCs, we first examine the azimuthal distribution of TC rain rate (see Methods). As shown in Fig. 2, the rain rate is generally azimuthally symmetric (or slightly asymmetric) in slow-moving TCs. For fast-moving TCs, however, the rain rate distribution is highly asymmetric, with a clear front and front-left maximum in the northern hemisphere (or front and front-right in the southern hemisphere), which is consistent with the results from previous work^21,22^. In TCs, strong convergent upward motion is likely the primary contributor to higher rain rates^21–23^. The convergence at 850 hPa and the vertical velocity at 500 hPa (Fig. 2 and Supplementary Fig. 4a, b) from the ERA5 dataset^18^ both strengthen with TC translation speed and have a maximum ahead of the TC. Similar distributions can also be found in each individual TC basin (Supplementary Fig. 5). In other words, the increase in rain rate with translation speed is driven by stronger convergence, and hence stronger rising motion, ahead of the TC (front and front-left maximum in the northern hemisphere, and front and front-right maximum in the southern hemisphere). However, the question is why fast-moving TCs have such an increase and an asymmetric variation in convergent rising motion.

In general, the distinct inflow layer in a typical TC extends from the surface to a height of ~2 km (lower troposphere), and the strongest inflow usually occurs near the surface^24^. We, therefore, investigate the azimuthal distribution of the radial wind (relative to the TC center; see Methods) at 10 m with TC translation speed. As shown in Fig. 2 and Supplementary Fig. 5, the radial wind strengthens in fast-moving TCs, especially in front of the TC. The net inflow (the average inflow over the TC region; see Methods) also strengthens generally with TC translation speed (Supplementary Fig. 4c). The large inflow ahead of the TC can enhance the convergent upward motion in fast-moving TCs (Fig. 2 and Supplementary Fig. 5). Therefore, the net inflow increase with TC translation speed is one possible contributing factor. But why does the inflow increase with translation speed, especially ahead of the TC?

Chan & Gray^25^ found that TC translation speed is usually greater than the surrounding flow, particularly in the surrounding lower-tropospheric flow around a TC (their Figs. 13, 14). As the TC rainfall-related convergence occurs mainly in the lower troposphere, we explore the relationship between the surrounding lower-tropospheric flow (see Methods) and TC translation speed. Results show that the surrounding lower-tropospheric flow is slower than the TC translation speed (Supplementary Fig. 4d), and the relative speed (TC translation speed minus the surrounding lower-tropospheric flow) increases significantly with translation speed (Supplementary Fig. 4e). Considering the continuity of the airflow, the increase in relative speed means that the growing airflow in the front of the TC enters to the TC region, superimposed on the inflow of the TC secondary circulation. This explains why the inflow is concentrated mostly in front of the TC and increases with translation speed.

### Vertical wind shear and tropical cyclone rain rate

**Fig. 3:**
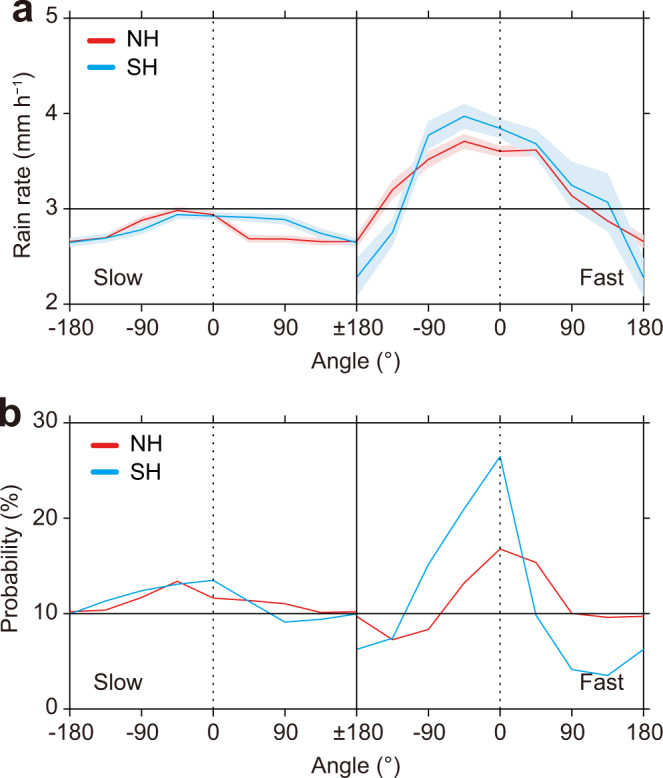
Azimuthal distributions of two variables with different vertical wind shear (VWS) directions. Left and right panels in each sub-figure represent **a** tropical cyclone (TC) rain rate and **b** the probability of VWS in slow- and fast-moving TCs, respectively. The *x* axis is the difference between the TC motion direction and the shear direction, “0” represents that the directions of VWS and TC motion are the same, “±180” represents the directions of VWS and TC motion are opposite, and clockwise is positive. Colors are for Northern Hemisphere (NH, red) and Southern Hemisphere (SH, blue), and shaded areas in **a** represent the standard error.

It is well known that the asymmetry of TC rainfall is greatly affected by vertical wind shear (VWS)^26–29^. A TC in a sheared environment is gradually tilted because of the different advection in the upper and lower layers, and TC rainfall is concentrated mainly in the downshear-left in the NH (or downshear-right in the SH) of the VWS. Because TC intensity and rain rate distribution can be influenced by the magnitude of VWS^26,27,29^, we first examine the change in TC rain rate under weak (VWS < 5 m s^–1^) and strong (VWS > 10 m s^–1^) VWS conditions. The result shows that the rain rates under both weak and strong VWS increase significantly with translation speed (Supplementary Fig. 6), which means that the magnitude of VWS has no obvious influence on the increase in TC rain rate with translation speed. Then, we investigated the impact from the direction of VWS. In fast-moving TCs, the maximum TC rain rate ahead of the TC may also be related to the larger probability of forward VWS (Fig. 3 and Supplementary Fig. 7; note: forward VWS means that the VWS contains a component towards the direction of TC motion; similarly, backward VWS means that the VWS contains a component towards the rear of the TC).

The results show that the average TC rain rate is also affected by the VWS direction, especially in a fast-moving TC. With forward VWS, the average TC rain rate increases more obviously with translation speed, but it is less obvious with backward VWS (Supplementary Fig. 8). In general, TC motion and VWS can cause the maximum TC rain rate to appear respectively in the front left of TC center and downshear left of VWS (i.e., influence area). Under the forward VWS condition, TC motion and VWS have a similar direction and their influence areas basically superimpose on each other, which is conducive to the formation of deep convection, and hence an increase in TC rain rate appearing in the front left of TC center. While, under the backward VWS condition, their influence areas do not overlap, which is unfavorable for the deep convection and the stronger rain rate, so that, the rain rate is relatively weaker. In a fast-moving TC, the rain rate is larger with forward VWS, combined with the larger proportion of forward VWS (Fig. 3), which can increase the average TC rain rate. Thus, the large probability of forward VWS is another contributor to the increased TC rain rate with translation speed. All TC basins, except the eastern North Pacific, have similar characteristics (Supplementary Fig. 7). In the eastern North Pacific, the larger probability of backward VWS leads to the suppression of the average TC rain rate increase with translation speed, which is the reason for the smaller rain rate in the fast-moving TCs than that in the other basins (Supplementary Fig. 2g and Supplementary Table 1).

However, why does the probability of forward WVS increase in fast-moving TCs? In general, the probability of fast-moving TCs increases at higher latitudes in the region of westerlies (Supplementary Fig. 9), where TCs move northeastward (or southeastward in the southern hemisphere) with strong westerly shear^24^, which increases the probability of forward VWS in fast-moving TCs.

### Impacts from tropical cyclone intensity

Changes in TC intensity can also affect TC rain rate. A stronger TC usually produces a higher rain rate^21,31^. As shown in Supplementary Fig. 10a, the average TC intensity increases significantly when the translation speed is <10 kt, which makes a positive contribution to the increase in TC rain rate. However, because a large translation speed tends to destroy the TC structure and suppress TC intensification^8^, the average TC intensity decreases when the translation speed is >10 kt (especially between 13 and 18 kt), which may make a negative contribution to the rain rate increase with translation speed (Fig. 1a, b and Supplementary Fig. 2a, b), as well as the convergent upward motion and the net inflow (Supplementary Fig. 4a–c). If we exclude the influence from TC intensity (see Methods), we still find that TC rain rate increases significantly with translation speed (Supplementary Fig. 11).

## Discussion

**Fig. 4:**
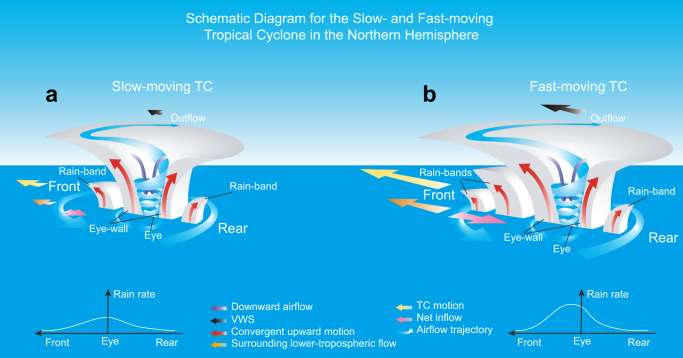
Schematic diagrams of the translating tropical cyclone (TC). **a** The slow-moving TC, **b** the fast-moving TC. The legend of colored arrows is listed at the lower of this figure. The white curves are the schematic diagram for the front-rear section of the rain rate composite of the slow- and fast-moving TCs. The schematic diagrams under forward and backward vertical wind shear (VWS) are shown in Supplementary Fig. 12.

The physical mechanisms behind the robust increase in TC rain rate with translation speed may be explained as a combination of two processes (Fig. 4 and Supplementary Fig. 12). Firstly, when a TC moves faster than the surrounding lower-tropospheric flow, the relative speed-induced net inflow, which increases with TC translation speed, and subsequent enhanced low-level convergence increase the convection in front of the TC. Secondly, forward VWS, the probability of which increases with translation speed and with proximity to the mid-latitude westerlies, leads to stronger convection to produce higher rain rates. This is because forward VWS enhances convection and rain rates in front of the TC as well, in the same region as the increased inflow increases it. Of course, TC rainfall is a complex process, and other factors may also affect the average TC rain rate with translation speed, so further work is needed.

In this study, the high-resolution ERA5 dataset is used to explore the possible physical mechanisms of TC rain rate increase with translation speed. Although this dataset is the best available, the reanalysis data could still be quite different from the actual situation in some cases so using the reanalysis data to investigate the physical mechanisms has some limitations. For example, Hersbach et al.^32^ noted that the mean absolute difference of ERA5 rain rate compared to TMPA/3B43 on the quasi-global scale (the domain from 50°S to 50°N) is 0.58 mm day^–1^. In this work, we found that the difference in the average TC rain rate between ERA5 and TMPA/3B42 can reach about 0.95 mm h^–1^, and the difference between ERA5 and IMERG is even 1.51 mm h^–1^. One of the most possible reasons may be that the TC intensity in ERA5 (maximum 10 m wind speed near the TC center) is significantly weaker than the observation (i.e., IBTrACS). Previous studies^33,34^ indicated that in ERA5 wind speeds within TCs do not exceed about 40 m s^–1^ (~78 kt), which means that the TCs in ERA5 rarely develop to be as intense as in the real atmosphere and thus cannot produce sufficient strong TC rain rate. Further, we have calculated the corresponding intensity of each observed TC sample from the ERA5 dataset, the result shows that the average TC intensity in ERA5 is ~20 kt weaker than the observations, and this bias increases gradually with the observed TC intensity (i.e., stronger TCs have a larger bias). In addition to the issue of the magnitude of the TC rain rate, the climate tendency of rain rate within the TC inner-core from ERA5 is opposite to that from the observations^31^, which is also an issue that deserves more concern. Therefore, it is important to improve the accuracy of the reanalysis data, and the large-scale and high-resolution observation data of other meteorological elements also need to be enhanced. In subsequent works related to this study, idealized TC numerical models can be used to explore and verify the changes in TC rain rate with translation speed and the important roles of TC inflow, vertical wind shear, and other possible contributing factors.

Previous studies have shown that the TC rainfall accumulation is proportional to the rain rate and inversely proportional to the TC translation speed^12–14^, but the effect of TC translation speed on the rain rate is usually ignored. Although we find a robust increase in TC rain rate with translation speed, the TC rainfall accumulation can still be larger for a slow-moving TC compared to a fast-moving TC if the rainfall contribution from the increased time of storm impact exceeds the decrease in average rainfall rate due to the slower translation speed. On the global scale, when a slow-moving TC accelerates to become a fast-moving one, the TC rainfall accumulation has a significant decrease, but its magnitude will be about a quarter (24%, Supplementary Table 1) greater than the unchanged rain rate condition. On the contrary, if a TC decelerates from fast to slow, the TC rainfall accumulation increases but its magnitude will be smaller. Therefore, if we do not consider the changes in TC rain rate with translation speed, the estimate of the TC rainfall accumulation may be off by up to a quarter. For category 3–5 TCs, it may even cause a 42% deviation (Supplementary Table 1).

TC rainfall-related disasters are caused not only by TC rainfall accumulation but also by high rain rates. According to TC model forecast evaluations, the accuracy of TC track forecasts has significantly improved in recent decades^35^. Therefore, combining the TC track forecasts (which impact the translation speeds) with this study, the short-term changes in TC rain rate can be estimated, which is beneficial to the short-term rainfall forecasts and disaster preparedness especially for accelerating or decelerating TCs. An increase in the translation speed of a landfalling TC also means that the amount of time before landfall will be shortened. Because the average TC rain rate increases with translation speed, more precisely, the probabilities of moderate and heavy rain are increased in a fast-moving TC. This situation poses a greater challenge in quickly responding to TC rainfall-related disasters, especially for underdeveloped regions and countries.

For projections of TC rain rate using climate models, researchers have suggested a robust increase, with a median of 14% for a 2 °C increase in temperature, under anthropogenic warming^36^. Recent studies^37–39^ have further suggested that the increase in TC rain rate with climate change often exceeds what might be expected from the Clausius-Clapeyron relation (~7% per °C). As a result, TC rainfall accumulation will likely increase further, which will seriously threaten TC-vulnerable areas. It should be emphasized that projections of TC rain rate are based mainly on the increasing water vapor and moisture convergence with climate change^36^. Our results provide a new perspective: the projected future decrease in TC translation speed^30,40^ may lead to a reduction in the increment of TC rain rate with climate change (i.e., the median <14%). Clearly, if the future TC translation speed in the future increases, which may cause an increase in the increment of TC rain rate (i.e., the median >14%), and also in the probability of TC moderate and heavy rain. However, there are still large uncertainties in the current projections of TC translation speed, but this also requires people to pay more attention, and further work is needed to verify the relevant conclusions.

## Methods

### Data

This study, which focuses on the period 2001–2020, uses the IBTrACS TC best-track dataset^16^, which contains the position of the TC center, maximum sustained wind speed, minimum pressure, translation speed, and TC direction, etc., at 3-hourly intervals. In this dataset, the TC translation speed and direction are calculated based on the position in latitude and longitude. IBTrACS contains the TC best-track records from different agencies. In this study, we use records from the following USA agencies: National Hurricane Center (NHC), Joint Typhoon Warning Center (JTWC), and Central Pacific Hurricane Center (CPHC).

TC rainfall is obtained from the IMERG final precipitation dataset^15^, which combines multi-satellite passive microwave precipitation estimates, microwave-calibrated infrared rain estimates, and rain gauge measurements to produce a rainfall estimate dataset with a 0.1° latitude × 0.1° longitude spatial resolution and a 30-min temporal resolution. The TMPA 3B42 dataset^17^ with 3-hourly intervals and 0.25° latitude × 0.25° longitude resolution is also used for verification. It should be noted that the instrument measurements of IMERG and TMPA are mostly the same, but the algorithms are different.

To explore the physical mechanisms, the 3-hourly ERA5 [the fifth generation ECMWF atmospheric reanalysis] dataset^18^, which includes total precipitation, wind field, divergence, and vertical velocity is also used.

### Tropical cyclone samples

From 2001 to 2020, there are a total of 124,601 instantaneous TC records (3-hourly) worldwide. Following the methods in Tu et al.^31^, we exclude the records of non-TC systems (such as extratropical systems, waves, disturbances, or others) and missing values. We also remove those records not at 00:00, 03:00, 06:00, 09:00, 12:00, 15:00, 18:00, or 21:00 UTC. We select TC activity within the range of 50°S-50°N. Supplementary Fig. 1a shows the frequency distribution of TC translation speed, which is concentrated mainly in the range of 1–20 kt (>95%; note: 1 kt ≈ 0.514 m s^–1^ ≈ 1.852 km h^–1^). The sample size of very fast-moving TCs (>20 kt) is relatively small, and those TCs are therefore excluded. Stationary TCs (0 kt) are also excluded. After these procedures, a total of 1898 TCs with 80,918 instantaneous TC rainfall samples (i.e., 3-hourly; note: these instantaneous TC rainfall samples including 21 samples in the South Atlantic are considered for the global scale and hemispheric-scale calculations unless otherwise stated) are obtained from the best-track data during the study period. To compare the difference in TC rain rate within slow- and fast-moving TCs more conveniently, we simply define those TCs with translation speed among 1–5 kt and 16-20 kt as the slow- and fast-moving TCs with 19,389 and 6249 samples on the global scale, respectively.

### Tropical cyclone rainfall radius

The most common method for separating TC rainfall from the precipitation product is intercepting rainfall within a fixed radius from the center of the TC, such as 500 km^10,21,27^, 444 km^41,42^, or, other special radii. There are several limitations when applying this method to study global TC rainfall because it does not consider the differences in TC intensity and basins. We, therefore, use the method described in Tu et al.^31^. That is, we calculate the all-pixels average rain rate (see the next section) in each 25 km annulus to obtain the radial distribution of each instantaneous TC rain rate, and further calculate the average radial curve of the TC rain rate with different TC intensities (i.e., tropical depressions (TDs, ≤34 kt), tropical storms (TSs, 35–63 kt), categories 1–2 (CAT12, 64–95 kt), and categories 3–5 (CAT35, ≥96 kt)) for each TC basin. We then define the distance from TC center to the location where the average rain rate >0.5 mm h^–1^ as TC rainfall radius. Supplementary Fig. 1b shows the different TC rainfall radii; hereafter, the area within the TC rainfall radius is defined as the TC region.

### Average tropical cyclone rain rate

Similar to Hu et al.^43^, we consider those pixels with rain rates ≥0.1 mm h^–1^ as TC rainfall (rain rate <0.1 mm h^–1^ is ignored). First, we count the average rain rate, including the rainy-pixels average (regarding the non-rainy pixels as NaN (not a number, uncounted)) and all-pixels average (regarding the non-rainy pixels as zero, counted) within the TC region of each TC sample, and then calculate the average rain rate with different TC translation speeds.

Changes in the average TC rain rate (note: the term “rain rate” represents the rainy-pixels average in the main text and Methods unless otherwise stated) are calculated globally (Fig. 1a), in both hemispheres (Northern Hemisphere, NH; Southern Hemisphere, SH, Fig. 1b, c), in six TC basins (the western and eastern North Pacific (WNP and ENP), North Atlantic (NA), South Indian Ocean (SI), South Pacific (SP), and North Indian Ocean basins (NI), Supplementary Fig. 2f–k), at different TC intensities (i.e., TDs, TSs, CAT12, and CAT35, Supplementary Fig. 2l–o), over the ocean and land (or landfalling before and landfalling after, Supplementary Fig. 2p, u), and also in different latitudinal belts (5–15°: the latitudinal belts of 5–15°S and 5–15°N; same with 15–25°, 25–35°, 35–45°, Supplementary Fig. 2q–t). The all-pixel averages on the global scale (Supplementary Fig. 2a) and hemisphere scale (Supplementary Fig. 2b, c) are calculated. In addition, changes in the TC rain rate from TMPA and ERA5 are also exhibited in Supplementary Fig. 2d, e.

To compare the difference in TC rain rate within slow-moving (1–5 kt) and fast-moving TCs (16–20 kt), we use the fitted linear regression line of the rain rate to simply estimate the rate of change (details in statistical information). The differences (in percentage) are shown in Supplementary Table 1.

### Tropical cyclone rain rate probability

In order to evaluate the relative contributions of the lower or higher rain rate in producing the increase shown in Fig. 1 and Supplementary Fig. 2, the anomalies of the TC rain rate probability are calculated. These anomalies are defined as the actual probability minus the mean probability of a given rain rate for different TC translation speeds. The results on a global scale are shown in Supplementary Fig. 3a. The results for the two hemispheres, different TC intensities, and individual TC basins are shown in Supplementary Fig. 3b–m.

### Azimuthal distribution of tropical cyclone rain rate

To evaluate the azimuthal distribution of TC rain rate with different TC translation speeds, we have rotated all the fields (the rain rate, vertical velocity, divergence, wind field, etc.) around the TC center to be relative to the translation direction of all TC samples. For example, as shown in Supplementary Fig. 1d, for a TC moving westward (in geographic coordinates, 0° points to north, so the angle of TC motion is 270°), we transform the new coordinates to the westward as 0° (rotating 270° anticlockwise).

The TC rain rate is divided into 36 bins around the TC center, and then the average value of each bin is calculated to obtain the azimuthal distribution of the TC rain rate. The azimuthal distribution of TC rain rate from IMERG is calculated. Figure 2 and Supplementary Fig. 5 show the standardized values (with fast- and slow-moving TCs standardize together) of the azimuthal distribution rain rate in the two hemispheres and six TC basins. Specifically, we assume that *X* = *(x*_*1*_*, x*_*2*_*… x*_*36*_*)* and *Y* = *(y*_*1*_*, y*_*2*_*… y*_*36*_*)* represent the azimuthal curves of slow- and fast-moving TCs. Then, we can get one sequence (*X,Y; i.e., x*_*1*_*, x*_*2*_ *… x*_*36*_*, y*_*1*_*, y*_*2*_ *… y*_*36*_*)* for the standardization analysis.

### Convergent upward motion

In this study, we focus on the variation in the vertical velocity at 500 hPa and the divergence at 850 hPa within the TC region from ERA5 dataset, which are also calculated as the average and the azimuthal distribution with TC translation speed (Fig. 2, Supplementary Fig. 4a, b). The method used here is the same as that for the azimuthal curves of rain rate. Note that for visual convenience, we have taken the opposite sign of “div850” and “w500” in Fig. 2 to make it consistent with the fluctuation of the rain rate.

### Low-level inflow

We use the wind field at 10 m within the TC region to investigate how the low-level inflow of TCs changes with translation speed. Similar to calculating the azimuthal rain rate, we calculate the radial component (inward is positive) of the 10 m wind field (note: the TC translation speed in the wind field at 10 m is removed, i.e., the parallel component along the TC motion of wind field minus the TC translation speed), and the results are shown in Fig. 2. For a fast-moving TC, there is the inflow in front of the TC but outflow near the rear. We, therefore, calculate the average (i.e., low-level net inflow) over the TC region (Supplementary Fig. 4c).

### The surrounding lower-tropospheric flow

Chan & Gray^25^ used the average wind speed within a 5–7° latitudinal radial band from the TC center as the speed of the surrounding flow. Here, to better match our research (TC rainfall-related convergence is occurring in the lower troposphere), we modify their method as follows. First, we calculate the average wind speed at 850 hPa within an annulus of 200 km outside the TC region. Further, this average wind speed is divided into a perpendicular component and a parallel component to the TC translation direction. Here, we define the parallel component of the average wind speed as the surrounding lower-tropospheric flow (Supplementary Fig. 4d), while the perpendicular component is relatively small and so is ignored. The relative speed is defined as the TC translation speed minus the surrounding lower-tropospheric flow of the TC (Supplementary Fig. 4e). The TC speed relative to the surrounding lower-tropospheric flow increases with translation speed so that the inflow ahead of the TC increases with translation speed. The relative wind speed at 10 m also increases clearly with translation speed (Supplementary Fig. 4f). These processes also enhance the convergent upward motion ahead of a fast-moving TC.

### Vertical wind shear

In this study, the VWS is defined as the average wind vector difference between 200 hPa and 850 hPa within the TC region of each TC sample, i.e.,

1 $${VWS}=\,\sqrt{{({U}_{200}-{U}_{850})}^{2}+{({V}_{200}-{V}_{850})}^{2}}$$

where *U* and *V* are the zonal and meridional mean of the wind speeds, the subscripts represent the different pressure levels. To explore the impact of the magnitude of VWS on the relationship between TC rain rate and translation speed, we have calculated the distributions of TC rain rate with different translation speeds under the weak (VWS < 5 m s^–1^) and strong (VWS > 10 m s^–1^) VWS conditions. The result is shown in Supplementary Fig. 6. Further, we calculate the direction of VWS (the angle of TC motion direction is 0°, and clockwise is positive) of all TC samples. Changes in the average TC rain rate with the angle of VWS are counted. Considering the sample sizes of VWS at different angles, we have counted the average TC rain rate and the probability of VWS in a 45°-bin (8 bins around the TC center), with results shown in Fig. 3.

For the forward VWS (note: forward VWS contains a component towards the direction of TC motion, and similarly, backward VWS contains a component towards the rear of the TC), the TC rain rate increases, especially for fast-moving TCs. To confirm superposition of TC motion and VWS on rain rate change, we simply calculate the change in average TC rain rate with translation speed under forward and backward VWS, respectively. The results are shown in Supplementary Fig. 8. Additionally, the schematic diagram for a translating TC under forward and backward VWS are also shown in Supplementary Fig. 12.

### Latitudinal change in tropical cyclone samples

We investigated the geographical distribution of TC samples at different latitudes, and then counted the percentage of TC sample sizes in each 5° latitudinal bin, with results shown in Supplementary Fig. 9.

### Tropical cyclone intensity

Observational evidence reveals that TC rain rate increases with TC intensity^19,29^. Similar to calculating the average TC rain rate and other factors, the average TC intensity change with translation speed are obtained (Supplementary Fig. 10a). To exclude the effect of TC intensity, we calculate the average rain rate with translation speed for the same TC intensity. Supplementary Fig. 10b shows the sample size distribution of TC intensity in a 10 kt bin. We selected samples with TC intensity of 25–34 kt, 45–54 kt, 65–74 kt, and 85–94 kt for statistics. The magnitude of the change in average TC intensity with translation speed is <1 kt, so the contributions from TC intensity could be excluded. The results with the same TC intensity are shown in Supplementary Fig. 11.

### Statistical information

Linear trends are estimated using simple linear regression of the TC-related variables with TC translation speed. Shaded areas in Fig. 1 and Supplementary Figs. 2, 4, 6, 8a, 8b, 10a, 11 are the two-sided 95% confidence bounds. Shaded areas in Figs. 2, 3a represent the standard error. The percentages in Supplementary Table 1 are calculated by finding the difference between the average of the first and last five points (i.e., 1–5 kt and 16–20 kt, which represent slow-moving TCs and fast-moving TCs, respectively) of the fitted linear regression line and then dividing by the average of the first five points. In this work, the significance of linear trends is determined using a two-tailed *t* test (with 18 degrees of freedom). It should also be noted that all the growth rates of TC average rain rate with TC translation speed listed in Supplementary Table 1 are significant at the 99% confidence level.

## Supplementary information

Supplementary Information

## Data Availability

TC best-track data are taken from the IBTrACS v4 dataset (https://www.ncdc.noaa.gov/ibtracs/). TC-related rainfall data are obtained from the IMERG Final Precipitation L3 Half Hourly 0.1 degree × 0.1 degree v06 dataset (https://earthdata.nasa.gov/), and the 3-hourly TMPA 3B42 v7 dataset (https://disc.gsfc.nasa.gov/datasets/TRMM_3B42_V7/summary?keywords=TRMM_3B42). Contributing factors to TC rain rate are taken from ERA5 (https://cds.climate.copernicus.eu/cdsapp#!/home). These datasets are publicly available. Source data are provided with this paper.

## Source data

Source Data

## Code availability

All the codes are available from the first author (S.T., email: shifei_tu@126.com) upon request.
