# Supplementary material for: Increase in tropical cyclone rain rate with translation speed
Source: Nat Commun. 2022 Nov 28;13:7325. doi: 10.1038/s41467-022-35113-8 (PMC9705401; doi:10.1038/s41467-022-35113-8)
Supplement: Supplementary file 1 — Supplementary Information [file 41467_2022_35113_MOESM1_ESM.pdf]

Supplementary Information

Increase in Tropical Cyclone Rain Rate with Translation Speed

Shifei Tu[1,2,3], Johnny C. L. Chan[4,5,6*], Jianjun Xu[1,3*], Quanjia Zhong[7], Wen Zhou[4,8], Yu Zhang[1,2]

[1] China Meteorological Administration-Guangdong Ocean University (CMA-GDOU) Joint Laboratory for Marine Meteorology & South China Sea Institute of Marine Meteorology, Guangdong Ocean University; Zhanjiang, China.

[2]College of Ocean and Meteorology, Guangdong Ocean University; Zhanjiang, China.

[3]Shenzhen Institute of Guangdong Ocean University; Shenzhen, China.

[4]Low-Carbon and Climate Impact Research Centre, School of Energy and Environment, City University of Hong Kong; Hong Kong, China.

[5]Shanghai Typhoon Institute, China Meteorological Administration, Shanghai, China.

[6]Asia-Pacific Typhoon Collaborative Research Center, Shanghai, China.

[7]State Key Laboratory of Numerical Modeling for Atmospheric Sciences and Geophysical Fluid Dynamics (LASG), Institute of Atmospheric Physics, Chinese Academy of Sciences; Beijing, China.

[8]Department of Atmospheric and Oceanic Sciences, Institute of Atmospheric Sciences, Fudan University; Shanghai, China.

*Corresponding authors. Email: johnny.chan@cityu.edu.hk & jxu@gdou.edu.cn

This PDF contains Supplementary Table 1 and Supplementary Figures 1-12.

Supplementary Table 1 | Statistics of tropical cyclone (TC) rain rate with TC translation speed for various regions, TC intensities, and latitudinal belts. "Growth rate" is the linear trend of rain rate change with TC translation speed, "P-value" represents the significance of these linear trends, and "Difference" represents the differences in average rain rate between fast- and slow-moving TCs (i.e., 1-5 kt and 16-20 kt, respectively; see Methods). The growth rates and differences are calculated globally, in the two hemispheres (NH: Northern Hemisphere, SH: Southern Hemisphere), in individual basins (WNP: western North Pacific, ENP: eastern North Pacific, NA: North Atlantic, SI: South Indian Ocean, SP: South Pacific, NI: North Indian Ocean), over the ocean and land, and also with different TC intensities (i.e., TDs: tropical depressions, TSs: tropical storms, CAT12: category 1-2, and CAT35: category 3-5) and in different latitudinal belts (5-15°, 15-25°, 25-35°, 35-45°), 5-15° represents the latitudinal belts of 5-15°S and 5-15°N, and similarly for 15-25°, 25-35°, and 35-45°. The statistics of the rainy-pixels average and all-pixels average are both listed.

	**Rainy-pixels average**			**All-pixels average**		
	**Growth rate (mm h^{-1} per kt)**	**P-value**	**Difference (%)**	**Growth rate (mm h^{-1} per kt)**	**P-value**	**Difference (%)**
**Global**	0.044 ± 0.003	< 0.0001	24	0.029±0.003	< 0.0001	33
**NH**	0.042 ± 0.003	< 0.0001	22	0.028±0.004	< 0.0001	31
**SH**	0.056 ± 0.004	< 0.0001	30	0.034±0.003	< 0.0001	40
**WNP**	0.042 ± 0.004	< 0.0001	21	0.025±0.004	< 0.0001	27
**ENP**	0.033 ± 0.008	0.0003	19	0.030±0.005	< 0.0001	36
**NA**	0.042 ± 0.004	< 0.0001	23	0.027±0.004	< 0.0001	32
**SI**	0.069 ± 0.006	< 0.0001	37	0.042±0.004	< 0.0001	49
**SP**	0.051 ± 0.005	< 0.0001	29	0.031±0.003	< 0.0001	40
**NI**	0.033 ± 0.009	0.0017	16	0.030±0.006	0.0002	33
**Ocean**	0.046 ± 0.003	< 0.0001	24	0.030 ± 0.003	< 0.0001	33
**Land**	0.026 ± 0.004	< 0.0001	15	0.025 ± 0.003	< 0.0001	35
**TDs**	0.016 ± 0.002	< 0.0001	9	0.009±0.001	< 0.0001	13
**TSs**	0.031 ± 0.003	< 0.0001	16	0.016±0.003	< 0.0001	18
**CAT12**	0.068 ± 0.006	< 0.0001	33	0.051±0.004	< 0.0001	49
**CAT35**	0.094 ± 0.006	< 0.0001	42	0.064±0.005	< 0.0001	50
**5-15°**	0.033 ± 0.004	< 0.0001	17	0.026±0.003	< 0.0001	27
**15-25°**	0.056 ± 0.004	< 0.0001	30	0.040±0.004	< 0.0001	47
**25-35°**	0.070 ± 0.006	< 0.0001	44	0.046±0.003	< 0.0001	72
**35-45°**	0.055 ± 0.005	< 0.0001	41	0.017±0.005	0.0054	32

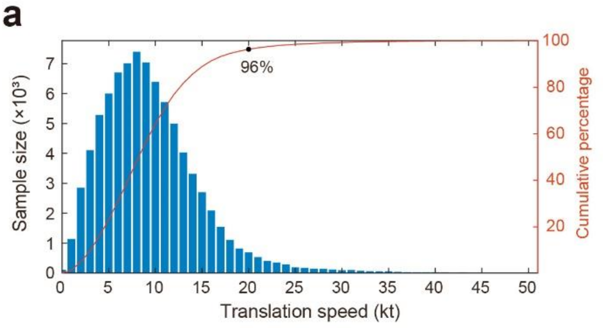

b

	TDs	TSs	CAT12	CAT35
WNP	550	550	500	475
ENP	350	350	325	350
NA	375	400	425	400
SI	450	475	450	450
SP	625	625	550	575
NI	475	475	425	425

TC rain radii (km)

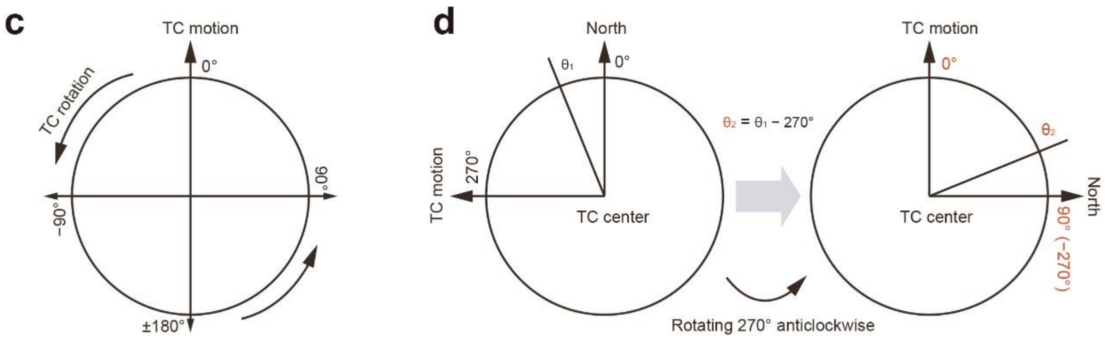

Supplementary Fig. 1 | Tropical cyclone (TC) sample size, TC rain radii, and schematic diagrams on TC coordinate rotation. a, Sample size of global TCs with different translation speed bins (histogram), and the cumulative percentage (red curve). **b**, TC rain radius with different TC intensities and TC basins. **c**, Schematic diagram of the angles for a translating TC. **d**, Schematic diagram showing coordinate rotation (see Methods).

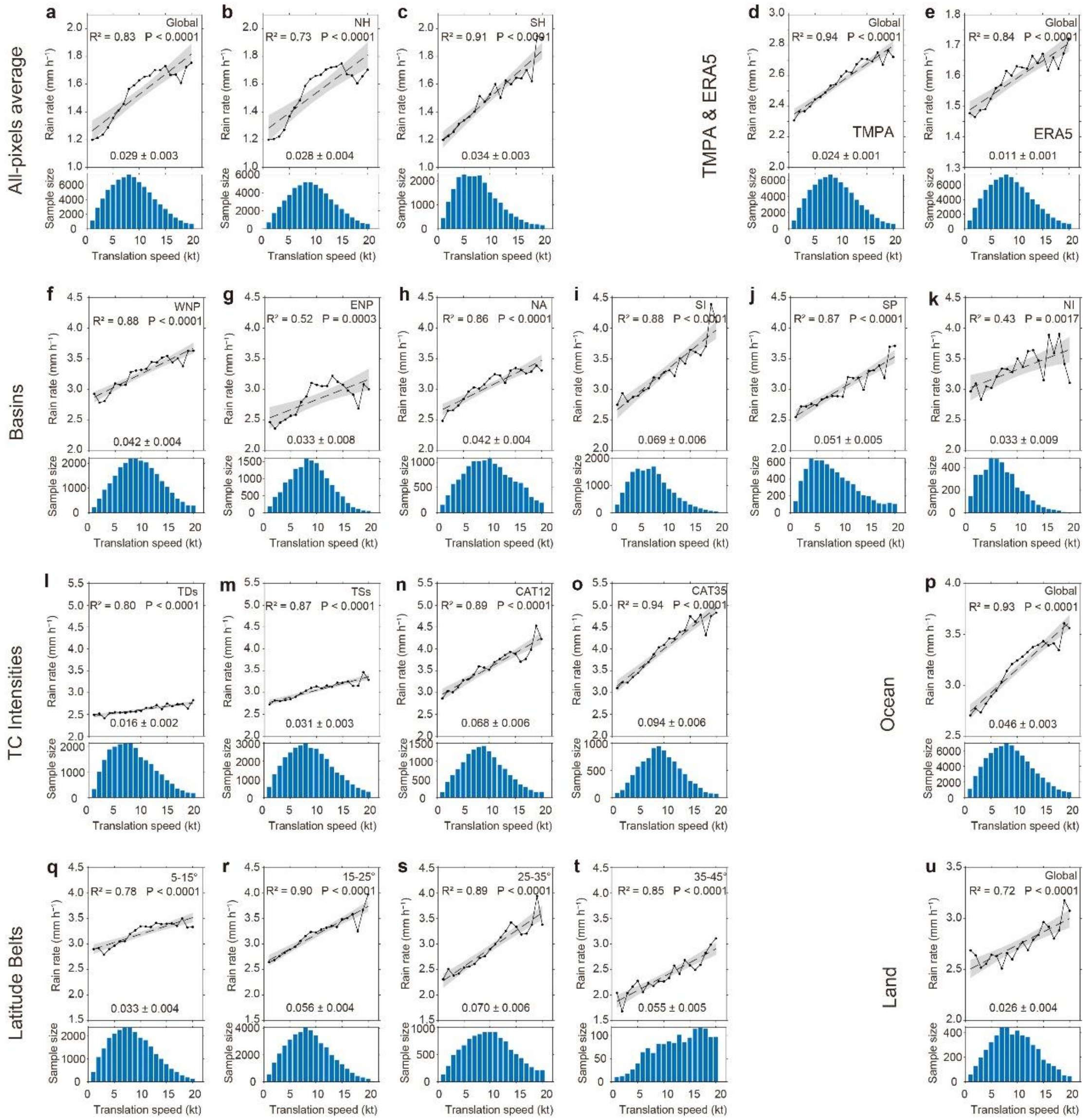

Supplementary Fig. 2 | Changes in average tropical cyclone (TC) rain rate with translation speed. The all-pixels average rain rate is calculated: **a**, Global, **b**, Northern Hemisphere (NH), and **c**, Southern Hemisphere (SH). The rainy-pixels average rain rate (globally) is obtained from **d**, the Tropical Rainfall Measuring Mission (TRMM) Multi-satellite Precipitation Analysis (TMPA), and **e**, the fifth-generation ECMWF (European Centre for Medium-Range Weather Forecasts) atmospheric reanalysis (ERA5) data. Rain rate of the rainy-pixels average change with translation speed in the six TC basins [**f**, western North Pacific (WNP), **g**, eastern North Pacific (ENP), **h**, North Atlantic (NA), **i**, South Indian Ocean (SI), **j**, South Pacific (SP), **k**, North Indian Ocean (NI)], with different TC intensities [**l**, tropical depressions (TDs), **m**, tropical storms (TSs), **n**, categories 1-2 (CAT12), and **o**, categories 3-5 (CAT35)], and **p**, over the ocean and **u**, over land, and in different latitudinal belts (**q**, 5-15°, **r**, 15-25°, **s**, 25-35°, **t**, 35-45°). The label 5-15° represents the latitudinal belts of 5-15°S and 5-15°N, and similarly for 15-25°, 25-35°, and 35-45°. The shaded area in all panels indicates the two-sided 95% confidence levels of

the trend, and the dashed lines represent the linear regression of the rain rate with TC translation speed. The numbers under the curve represent the growth rate. All linear trends are significant at the 99% confidence level. All average rain rates are obtained from the Integrated Multi-satellite Retrievals from the Global Precipitation Measurement (IMERG) final precipitation dataset except **d-e**, which are obtained from TMPA and ERA5 data. The bar chart in each sub-figure represents the distribution of TC samples.

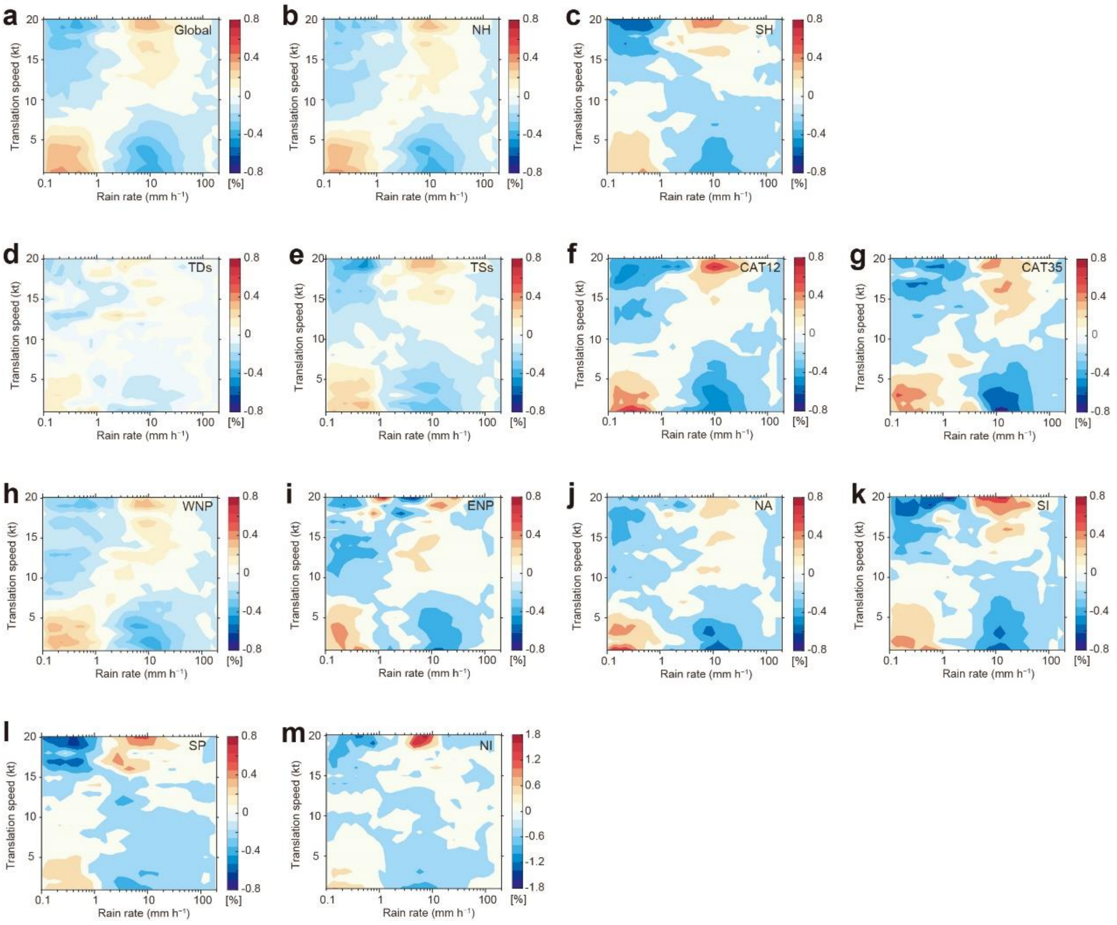

Supplementary Fig. 3 | Probability anomalies of tropical cyclone (TC) rain rate with translation speed. **a**, Global, **b**, Northern Hemisphere (NH), **c**, Southern Hemisphere (SH), different TC intensities [**d**, tropical depressions (TDs), **e**, tropical storms (TSs), **f**, categories 1-2 (CAT12), and **g**, categories 3-5 (CAT35)], and different TC basins [**h**, western North Pacific (WNP), **i**, eastern North Pacific (ENP), **j**, North Atlantic (NA), **k**, South Indian Ocean (SI), **l**, South Pacific (SP), **m**, North Indian Ocean (NI)]. Warm colors indicate the increased probability.

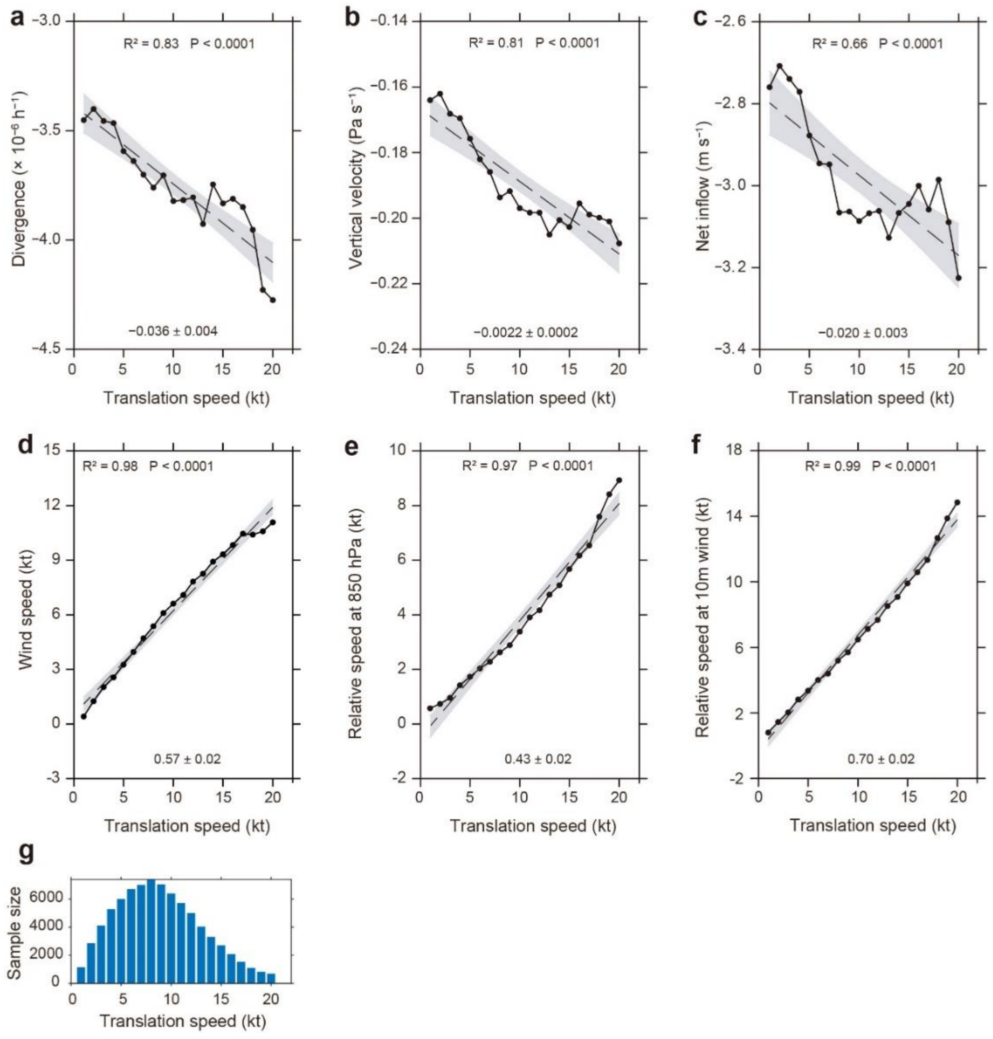

Supplementary Fig. 4 | Changes in the different variables with translation speed. **a**, Divergence at 850 hPa. **b**. Omega (i.e., vertical velocity) at 500 hPa. **c**, Low-level net inflow at 10 m. **d**, Surrounding lower-tropospheric flow (850 hPa, see Methods). **e**, Relative speed at 850 hPa. **f**, Relative speed at 10 m. **g**, Tropical cyclone (TC) samples distribution of **a-f**. All these different variables are calculated over the whole TC region. Definitions can be found in Methods. The shaded area in all panels indicates the two-sided 95% confidence levels of the trends, the dashed lines represent the linear regression of the rain rate with TC translation speed, and the numbers under the curve represent the growth rate. All linear trends are significant at the 99% confidence level.

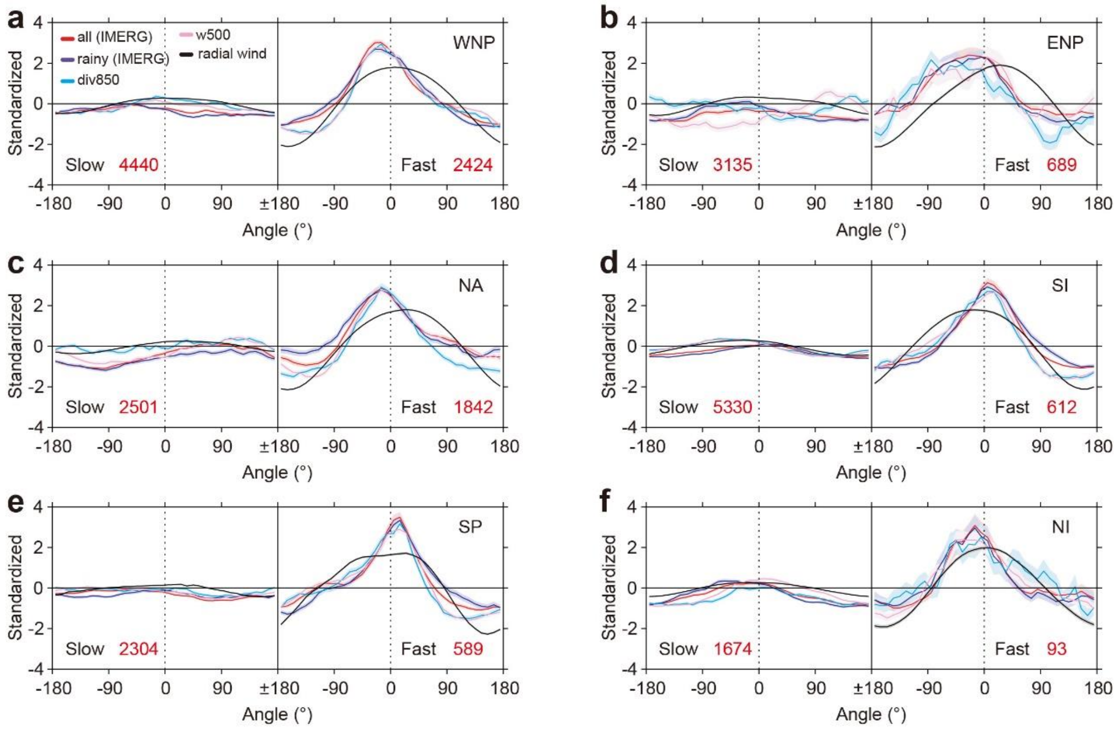

Supplementary Fig. 5 | Azimuthal distribution of different variables. Left and right panels in each sub-figure represent these variables in slow- and fast-moving tropical cyclones (TCs), respectively. **a**, western North Pacific (WNP), **b**, eastern North Pacific (ENP), **c**, North Atlantic (NA), **d**, South Indian Ocean (SI), **e**, South Pacific (SP), **f**, North Indian Ocean (NI). The x-axis represents different angles, as in the schematic diagram shown in Supplementary Fig. 1c; "0" represents the direction of TC motion, "±180" represents the reverse of TC motion, and clockwise is positive. Colors are for different standardized variables: all-pixels average (red) and rainy-pixels average (purple) in TC rain rate, vertical velocity at 500 hPa (pink, upward is positive), divergence at 850 hPa (blue, convergence is positive), and radial wind at 10 m (black, inflow is positive). Shaded areas in each sub-figure represent the standard error. Red numbers indicate the sample sizes used in each plot.

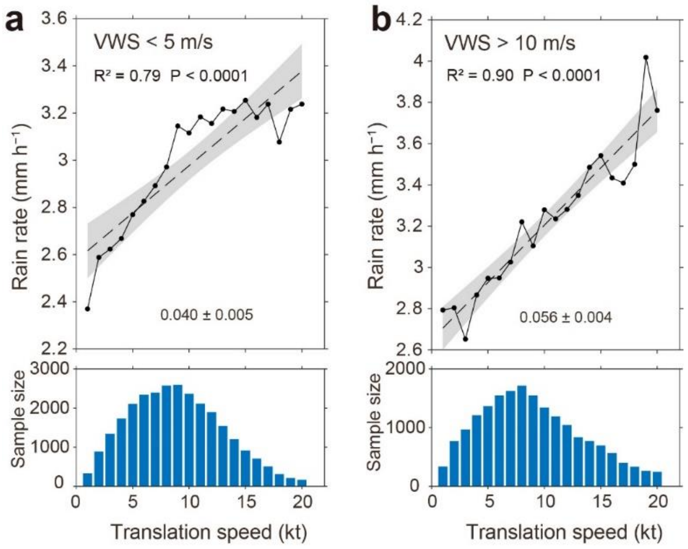

Supplementary Fig. 6 | Changes in average tropical cyclone (TC) rain rate with translation speed under the different VWS conditions. a, weak VWS (< 5 m/s), **b**, strong VWS (>10 m/s). The shaded area in all panels indicates the two-sided 95% confidence levels of the slopes, the numbers under the curve represent the growth rate, and the dashed lines represent the linear regression of the rain rate with TC translation speed. All linear trends are significant at the 99% confidence level. The bar chart in each sub-figure represents the distribution of TC samples.

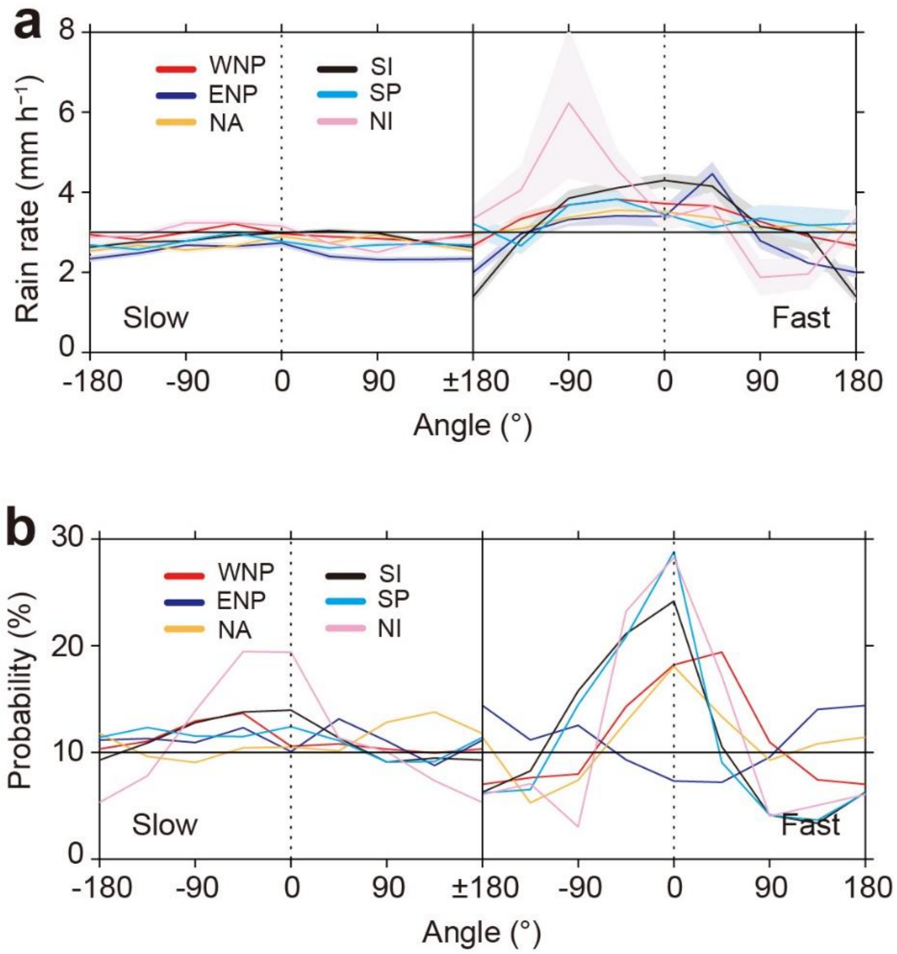

Supplementary Fig. 7 | Azimuthal distributions of two variables with different vertical wind shear (VWS) directions. a, tropical cyclone (TC) rain rate, and **b**, the probability of VWS. Left and right panels in each sub-figure represent these variables in slow- and fast-moving TCs, respectively. The x-axis is the difference between the TC motion direction and the shear direction, "0" represents that the directions of VWS and TC motion are the same, "±180" represents the directions of VWS and TC motion are opposite, and clockwise is positive. Colors represent the different TC basins: western Northern Pacific (WNP, red), eastern North Pacific (ENP, purple), North Atlantic (NA, orange), Southern Indian Ocean (SI, black), South Pacific (SP, blue), and North Indian Ocean (NI, pink). Shaded areas in (**a**) represent the standard error. The sample sizes of each plot are the same as in Supplementary Fig. 5.

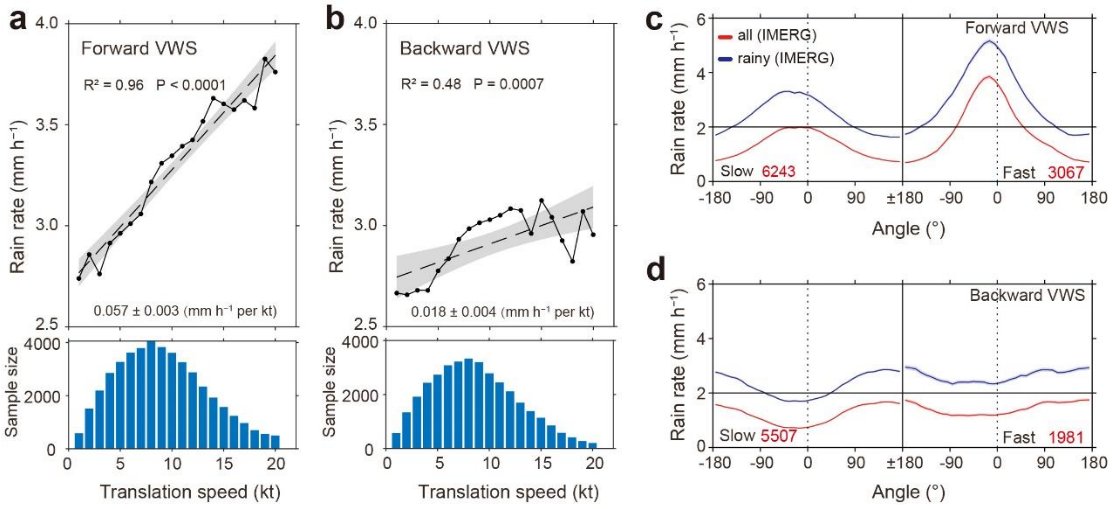

Supplementary Fig. 8 | Analysis of tropical cyclone (TC) rain rate and vertical wind shear (VWS). a, Changes in the average TC rain rate with translation speed under forward VWS, **b**, Same as **a**, but for backward VWS. The shaded areas in **a-b** indicate the two-sided 95% confidence levels of the trends, and the dashed lines represent the linear regression of the rain rate with TC translation speed. The linear trends in **a-b** are significant at the 99% confidence level, and the numbers under the curve represent the growth rate. The bar chart in **a-b** represents the distribution of TC samples. **c**, Azimuthal distribution of rain rate in the Northern Hemisphere (NH) from the Integrated Multi-satellite Retrievals from the Global Precipitation Measurement (IMERG) final precipitation under forward VWS, red for all-pixels average and blue for rainy-pixels average. **d**, Same as **c**, but for backward VWS. Left and right panels in **c-d** represent rain rate in slow- and fast-moving TCs, respectively; the x-axis represents different angles, as in the schematic diagram shown in Supplementary Fig. 1c; “0” represents the direction of TC motion, “±180” represents the reverse of TC motion, and clockwise is positive. Red numbers in **c-d** indicate the sample sizes used in each plot.

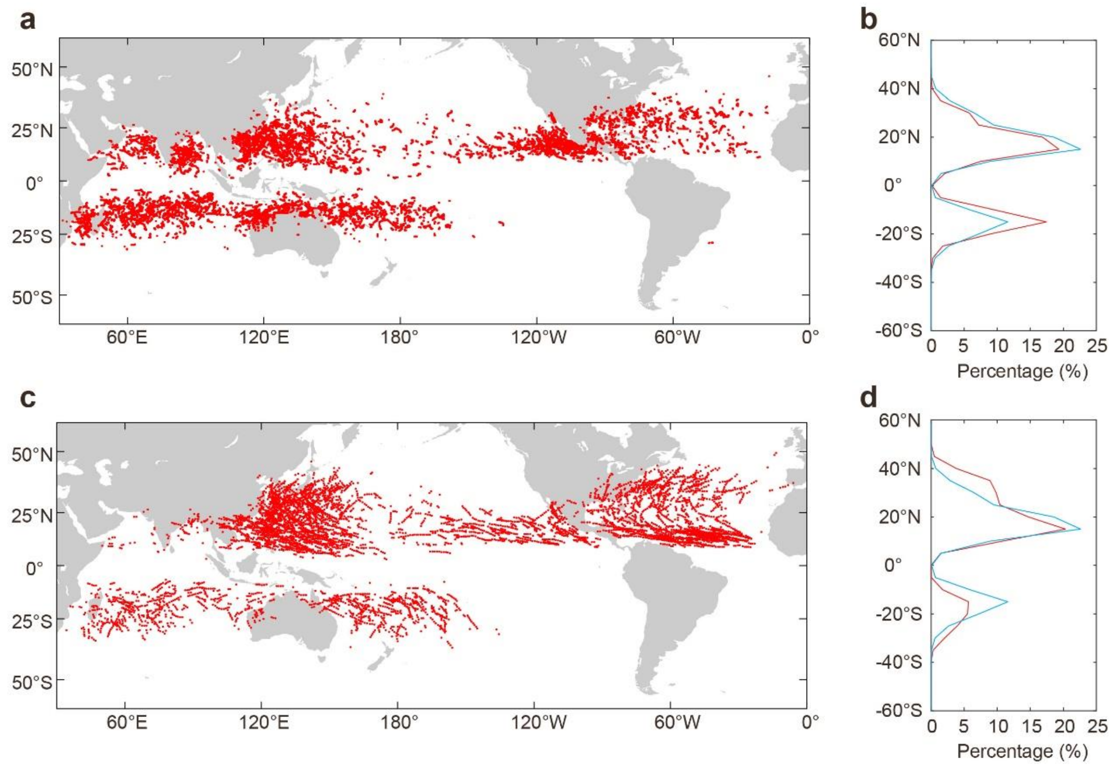

Supplementary Fig. 9 | Geographical distributions of tropical cyclone (TC) samples. **a**, Geographical distribution of slow-moving TCs. **b**, Probability distributions of slow-moving TC samples at different latitudes (red curve). **c**, Same as **a**, but for fast-moving TCs. **d**, Same as **b**, but for fast-moving TCs. Blue curves in **b** and **d** represent the probability distributions of all TC samples. Each probability is calculated in a 5° latitudinal bin.

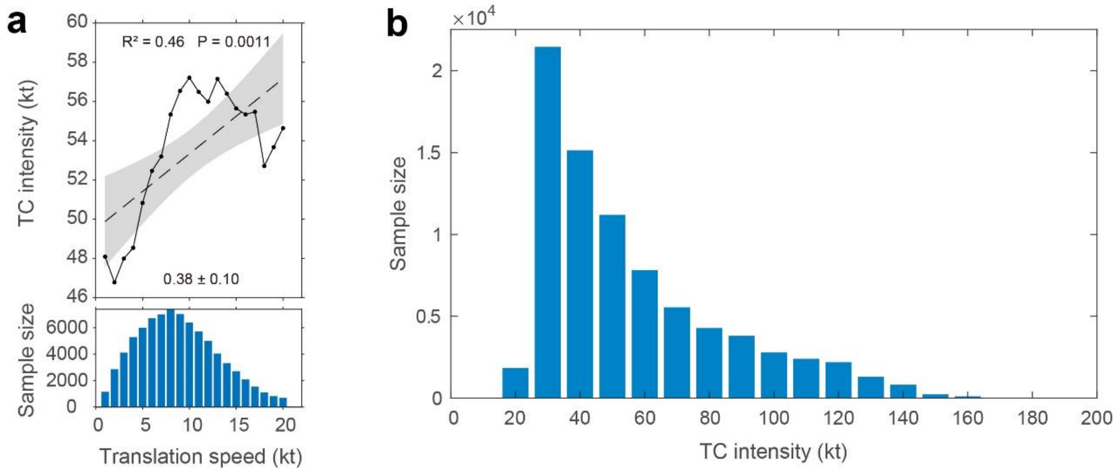

Supplementary Fig. 10 | Analysis of tropical cyclone (TC) intensity. a, Average TC intensity changes with translation speed. The shaded area indicates the two-sided 95% confidence levels of the trends, and the dashed lines represent the linear regression of the rain rate with TC translation speed. The numbers under the curve represent the growth rate. The bar chart represents the distribution of TC samples. **b**, The sample size distribution of TC intensity rounding into the 10 kt bins.

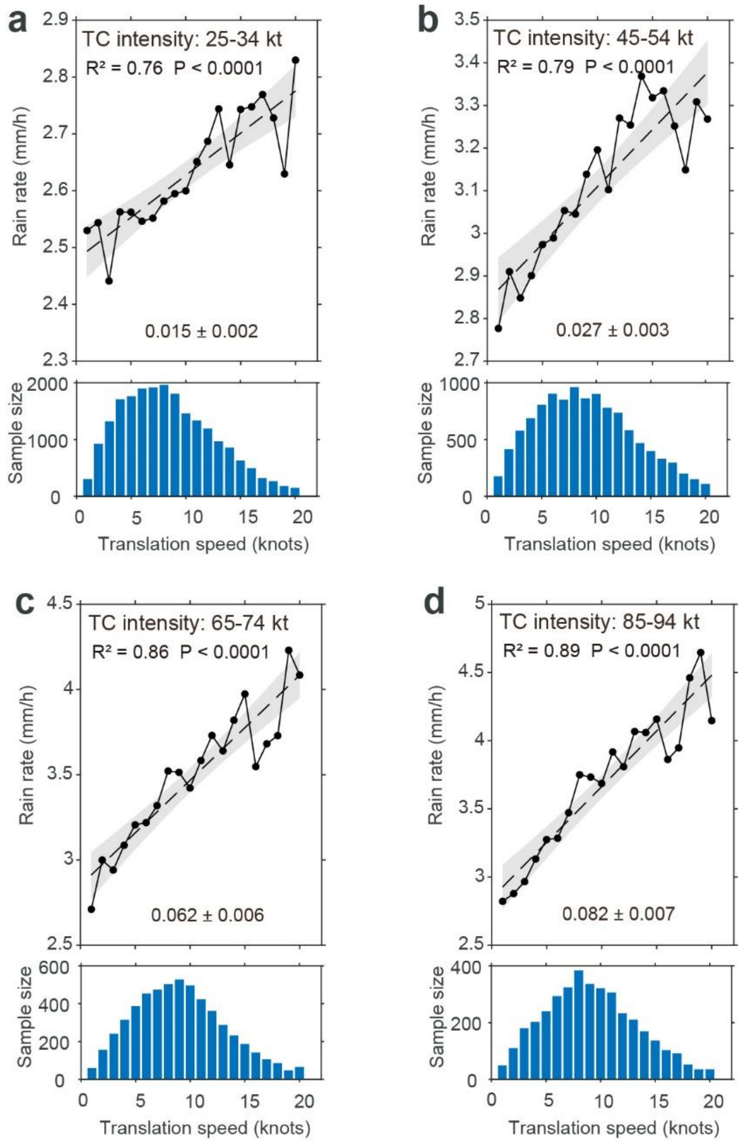

Supplementary Fig. 11 | Rain rate and influence factors of the same tropical cyclone (TC) intensity change with translation speed. The four columns from left to right indicate those samples with TC intensity of **a.** 25-34 kt, **b.** 45-54 kt, **c.** 65-74 kt, and **d.** 85-94 kt, respectively. TC rain rate from the Integrated Multi-satellite Retrievals from the Global Precipitation Measurement (IMERG) final precipitation data. The shaded areas indicate the two-sided 95% confidence levels of the trends, the dashed lines represent the linear regression of the rain rate with TC translation speed, and the numbers under the curve represent the growth rate. All the linear regressions are significant (99% confidence level). The bar chart in each sub-figure represents the distribution of TC samples.

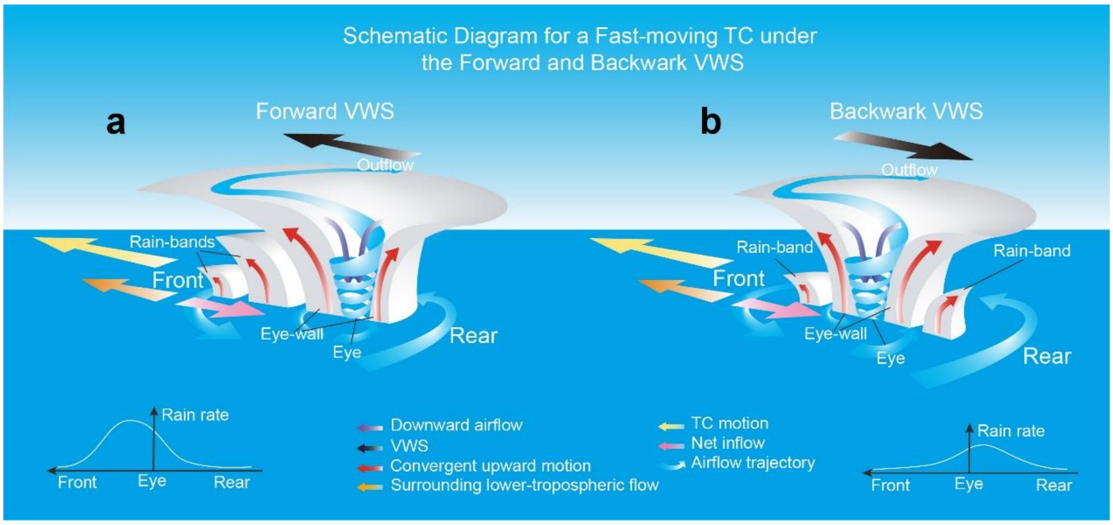

Supplementary Fig. 12 | Schematic diagrams of the fast-moving tropical cyclones (TCs). a, the forward vertical wind shear (VWS) condition, and **b**, the backward VWS condition. The legends of colored arrows are listed at the lower of this figure. The white curves are the schematic diagram for the front-rear section of the rain rate composite of the slow- and fast-moving TCs.
